# Driver drowsiness estimation using EEG signals with a dynamical encoder–decoder modeling framework

**DOI:** 10.1038/s41598-022-05810-x

**Published:** 2022-02-16

**Authors:** Sadegh Arefnezhad, James Hamet, Arno Eichberger, Matthias Frühwirth, Anja Ischebeck, Ioana Victoria Koglbauer, Maximilian Moser, Ali Yousefi

**Affiliations:** 1 Institute of Automotive Engineering, Graz University of Technology, 8010 Graz, Austria; 2 Neurable Company, Boston, MA 02108 USA; 3 Human Research Institute, Weiz, 8160 Austria; 4 Institute of Psychology, University of Graz, 8010 Graz, Austria; 5 Institute of Engineering and Business Informatics, Graz University of Technology, Graz, 8010 Austria; 6 Department of Computer Science Worcester Polytechnic Institute, 100 Institute Road, MA 01609 Worcester, USA; 7 Present Address: Vistim Labs Company, Salt Lake City, UT 84103 USA; 8 Chair of Department of Physiology, Medical University of Graz, 8036 Graz, Austria

**Keywords:** Neuroscience, Biomedical engineering, Mechanical engineering

## Abstract

Drowsiness is a leading cause of accidents on the road as it negatively affects the driver’s ability to safely operate a vehicle. Neural activity recorded by EEG electrodes is a widely used physiological correlate of driver drowsiness. This paper presents a novel dynamical modeling solution to estimate the instantaneous level of the driver drowsiness using EEG signals, where the PERcentage of eyelid CLOSure (PERCLOS) is employed as the ground truth of driver drowsiness. Applying our proposed modeling framework, we find neural features present in EEG data that encode PERCLOS. In the decoding phase, we use a Bayesian filtering solution to estimate the PERCLOS level over time. A data set that comprises 18 driving tests, conducted by 13 drivers, has been used to investigate the performance of the proposed framework. The modeling performance in estimation of PERCLOS provides robust and repeatable results in tests with manual and automated driving modes by an average RMSE of 0.117 (at a PERCLOS range of 0 to 1) and average High Probability Density percentage of 62.5%. We further hypothesized that there are biomarkers that encode the PERCLOS across different driving tests and participants. Using this solution, we identified possible biomarkers such as Theta and Delta powers. Results show that about 73% and 66% of the Theta and Delta powers which are selected as biomarkers are increasing as PERCLOS grows during the driving test. We argue that the proposed method is a robust and reliable solution to estimate drowsiness in real-time which opens the door in utilizing EEG-based measures in driver drowsiness detection systems.

## Introduction

Recent studies have shown that drowsiness is one of the major factors of road accidents that causes a large number of fatalities and monetary losses^1–4^. National Highway Traffic Safety Administration (NHTSA) announces that about 1.9% of total driving fatalities in 2019 (697 fatalities) were caused by drowsy drivers^5^. In another report, NHTSA estimated that in 2017, 91,000 police-reported crashes involved drowsy drivers that led to approximately 50,000 traffic injuries and 800 fatalities^6^. An assessment of the American Automobile Association (AAA) found that about 24% of drivers revealed been extremely drowsy while driving, at least once in the last month^7^. Furthermore, 14.5% of the drivers in the USA have been involved in at least one drowsiness-related traffic collision, according to a study carried out in 2008^8^. Some studies also showed that the level of drowsiness in automated driving is significantly higher than in manual driving^10–12^. Given all this evidence, the estimation of driver fatigue is essential for road safety and also future intelligent transportation systems require a vigilant driver for take-over requests from automated vehicles failing to perform safely.

Generally, three types of data have been used in the literature to design driver drowsiness detection systems: (1) vehicle-based^13,14^, (2) vision-based^15,16^, and (3) physiological data^17,18^. The literature suggests that physiological data such as EEG may be more appropriate than other systems to detect the onset of driver drowsiness^19,20^ specifically because vehicle-based and vision-based systems can be too late in warning the driver in the early stages of drowsiness, when there might still be time to prevent the accident. Critical signs of drowsiness such as yawning and head-nodding often appear before lateral displacement of the car and other non-physiological signs. Vision-based systems, while convenient, also suffer from robustness limitations in different light conditions and their performance can be significantly degraded when the drivers wear glasses or sunglasses^21,22^. Furthermore, data privacy can also be another issue for vision-based drowsiness detection systems which should be more studied in future research works.

Neural activities collected using EEG electrodes are widely exploited to classify and predict the different levels of driver drowsiness. There are many methods to produce these classifications and predictions, including a range of useful EEG layouts and machine learning techniques. For example, in designing a driver drowsiness detection system, Ma et al.^23^ used the Principal Component Analysis (PCA) technique and a deep neural network to extract features and predict instances of drowsiness using EEG data. Another study used features such as Higuchi and Petrosian fractal dimensions, and the logarithm of energy extracted from EEG as inputs to a Neural Network (NN) which is trained to classify the driver drowsiness^24^. Chen et al. used a similar NN-based classifier with different non-linear neural features extracted from subbands of the EEG signals using wavelet transformation^25^. There is also research suggesting single EEG channel recordings from the T7 electrode in the temporal lobe have predictive power to detect driver drowsiness. Wavelet transform has been used to extract features from this channel and classical classifiers have obtained satisfying performance for drowsiness classification^26^. Bajaj et al.^27^ proposed another EEG feature extraction methodology based on tunable Q-factor wavelet transformation. The extracted features were then interpreted by classifiers such as support vector machines and K-nearest neighbors to classify the driver’s vigilance as alert or drowsy. Yeo et al.^28^ also proposed a method based on a support vector machine classifier trained by several extracted frequency-domain features from EEG sub-bands. Independent Component Analysis (ICA) has been exploited in^29^ to extract the EEG sources where ICA models are designed for each alertness and drowsiness state. Results showed that ICA models are negatively and positively correlated with reaction speeds in the alertness and drowsiness states, respectively. The power spectrum of EEG sub-bands has been analyzed in^30^ and results showed Alpha and Theta band powers increase significantly during transition from alert to drowsy state. Budak et al.^31^ also proposed the ensemble majority voting of three deep networks that were trained using different EEG features to classify the vigilance state into two classes: awake and drowsy. On the contrary, a support vector machine-based posterior probabilistic model was proposed in^32^ that used the power of Theta, Alpha, and Beta sub-bands of EEG data and transformed the drowsiness level to any value between 0 and 1.

Aforementioned solutions show the promising classification of driver drowsiness using EEG-based neural features. For a system to be useful in predicting drowsiness before a subject is drowsy and accident prevention is possible, we can improve on this work by providing information on how the level of drowsiness changes as a function of neural activities. In other words, these existing methods are unable to detect the early stages of the drivers’ drowsiness, when drivers can be warned early enough to prevent impaired driving. We seek, with this paper, to solve this issue by developing a framework that characterizes the distribution of neural activities as a function of driver drowsiness. A scientifically established measure to assess driver drowsiness is the PERcentage of Eyelid CLOSure (PERCLOS)^33,34^. Therefore, we use this variable as the ground truth of the driver drowsiness and our proposed modeling framework predicts PERCLOS as a function of neural features. To resolve the issue of previous methods, a generative model for drowsiness tracking is proposed in this paper that provides a moment-to-moment assessment of PERCLOS. This method provides a posterior distribution of PERCLOS. Therefore, we can build other metrics like drowsiness level at a specific time or over a period as a function of the PERCLOS posterior distribution estimate. In other words, this method makes it possible to predict the trajectory of PERCLOS in the next multiple seconds which is an important factor to prevent accidents or create a timely countermeasure.

Alongside developing a real-time modeling solution to estimate driver drowsiness, we are interested in identifying neural biomarkers of drowsiness which may be useful to others studying drowsiness and needing reliable biomarkers. In the development of our modeling solution, we expand on the dynamical neural encoder-decoder modeling framework which has been successfully utilized in other applications such as extracting multi-dimensional auditory and visual stimulus-response correlations^35^, decoding neural recordings to predict speech^36^, reconstructing natural images using Bayesian decoder^37^, and decoding hidden cognitive states^38^.

In the extension of the dynamical encoder-decoder modeling framework in estimating PERCLOS, we provide a new model to characterize the temporal dynamics of PERCLOS. Using neural encoder models, we search for a subset of neural features encoding PERCLOS. We finally demonstrate how the state process for the PERCLOS and neural encoder models can be combined to estimate PERCLOS in real-time.

## Data collection and study procedure

### Apparatus

**Figure 1:**
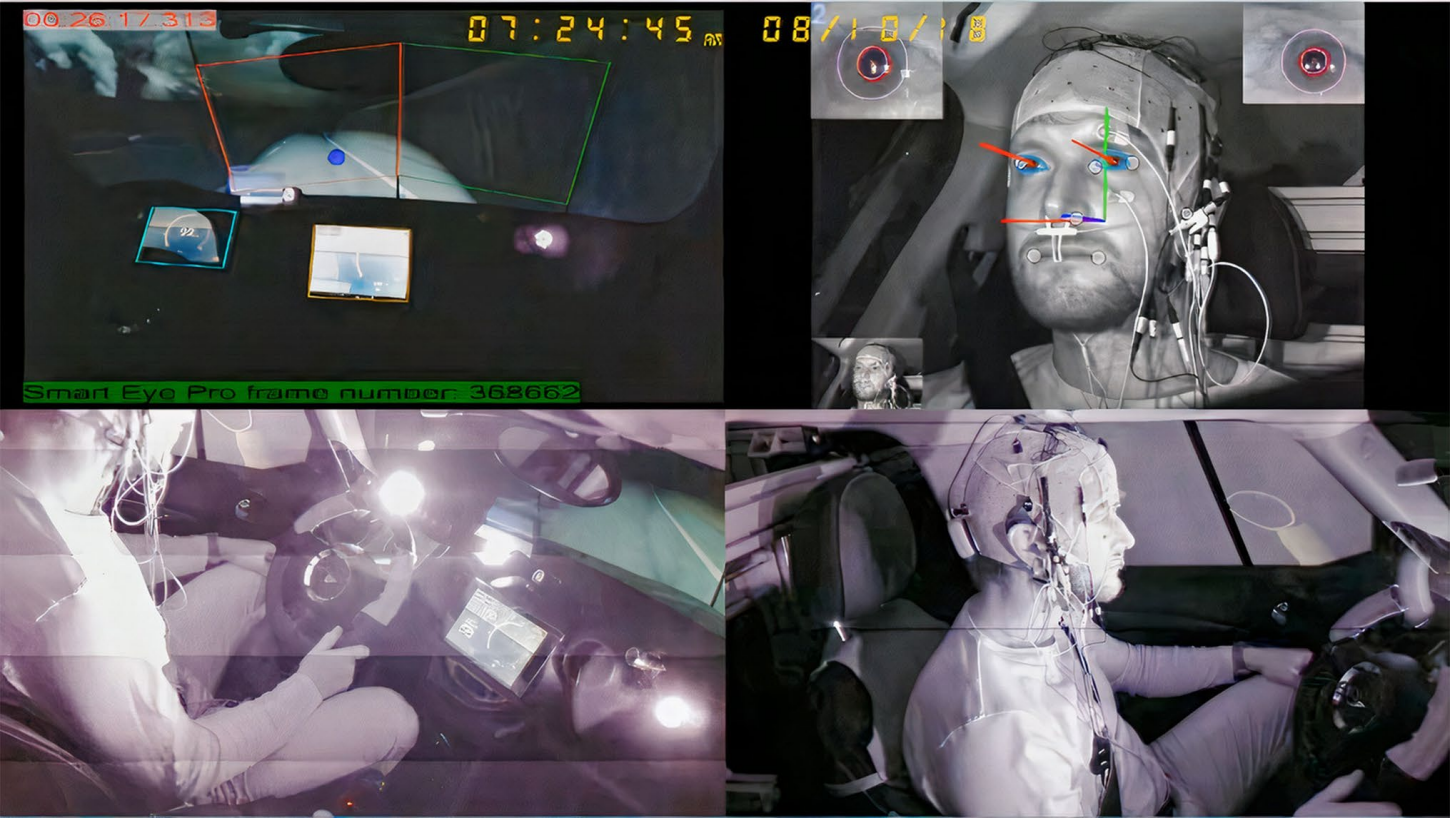
Four different views of the driver when he was conducting the test. The test road in a night drive and a dashboard instrument that shows the velocity of the vehicle in the simulated test track are shown in the left part. The output of the eye-tracking system that detects the eyelid movements and pupil diameter is presented in the right upper part (Reprinted from our previous study^14^; Copyright (2021), with permission from Elsevier, License No. 5058681457961. Informed consent was obtained from the driver to publish his image.

This study was carried out in a fixed-base driving simulator called Automated Driving Simulator of Graz (ADSG) at the Graz University of Technology (TU Graz), which is based on a full production vehicle. Visual cues are simulated by eight large LCDs, placed around the windshield and the left and right side windows, and one in the rear section of the car. Acoustic cues are simulated by a stereo sound system and several shakers, providing engine sound, background noise, and vibrations. The vehicle has an automatic gearbox, and drivers can control the car using a force feedback steering wheel and pedals. The realism of the simulator was validated with driving tests in previous projects^39^. Automated driving functions are implemented for longitudinal (by employing the adaptive cruise control) and lateral vehicle control (by employing lane-keeping assist). The driver information was limited to speed and indicator information, using a tablet PC. The driver can operate adaptive cruise control and lane-keeping assist systems with a touch screen located on the right side of the dashboard. For the present study, EEG channels are collected using an g.Nautilus Research® EEG cap (https://www.gtec.at/) and driver’s head position, eyelid movement, pupil diameter, and gaze direction are also measured with an infrared-based eye-tracking system called SmartEye® (https://smarteye.se/). Figure 1 shows four different views of a driver when he was performing the test.

### Driving tests procedure

In this study, drivers participated in two different driving modes: ‘manual’ and ‘automated’. In the automated mode, lane-keeping and cruise control systems adjust the vehicle’s lateral position and longitudinal speed in the test track, respectively. Drivers conducted a 30-minute highway driving test in each of these modes in two states including ‘rested’ and ‘fatigued’. In the rested state test, drivers were asked to stick to a full night’s sleep routine before the test and not diverge from their usual circadian cycle. For carrying out the fatigued state test, there were two choices. One choice was to stay awake for at least 16 hours continuously before starting the test procedure (resulting in a drive after at least 17 to 18 hours of wakefulness) and to take the test at their usual bedtime. Another choice was a sleep restriction of at least 50% (max. 4 hours of sleep) the night before the test. Overall, 92 drivers, balanced in age and gender participated in the four different driving tests described above. More information about the testing procedure can be found in our previous work^14^.

**Figure 2:**
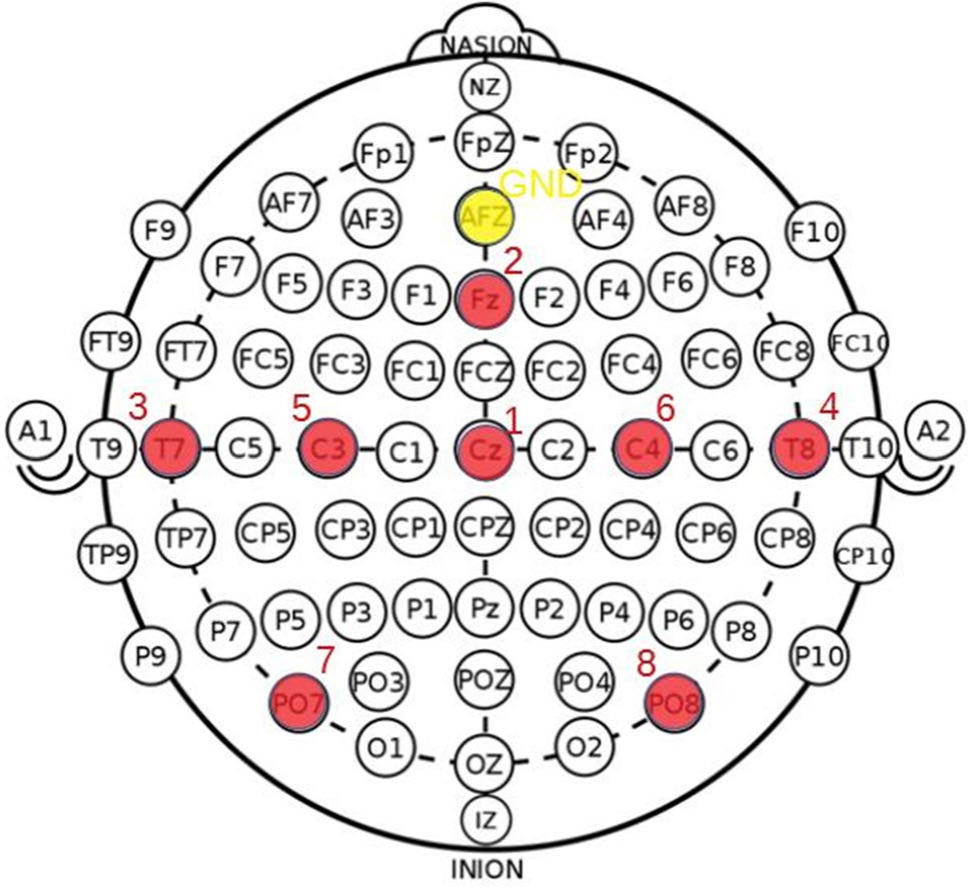
The positions of the EEG electrodes are shown by red circles. These electrodes cover the frontal lobe (by Fz), central lobe (by C3, C4, and Cz), temporal lobe (by T7 and T8), and parietal lobe (by PO7 and PO8). Two EOG electrodes (not shown here) were also placed in the lower and upper areas of the right eye to record eye movements. The ground electrode was placed in the AFZ position, marked by a yellow circle.

In this study, a data subsample of 18 driving sessions from 13 drivers (5 females and 8 males; age $44.5\mp 18.8$ years) that have various levels of PERCLOS and acceptable eyelid data quality have been selected to estimate the level of drowsiness in drivers. The study was conducted according to the guidelines of the Declaration of Helsinki, and approved by the Ethics Committee of Medical University of Graz (Code 30-409 ex 17/18, approved on 03.08.2018). Informed consent was obtained from all participants before the experiments. EEG signals were collected using gel electrodes, with a sampling frequency of 500 Hz, and 24-bit resolution. Eight electrodes have been used to collect EEG signals including Cz, Fz, T7, T8, C3, C4, PO7, and PO8. To capture eye movements, two EOG electrodes were attached above and below the right eye. The EOG signal was calculated as the difference between the two EOG electrodes data. The positions of the EEG electrodes along with the ground electrode (GND) placed in the AFZ electrode are marked by red and yellow circles in Fig. 2, respectively.

## Methodology

### Calculation of the actual PERCLOS

**Figure 3:**
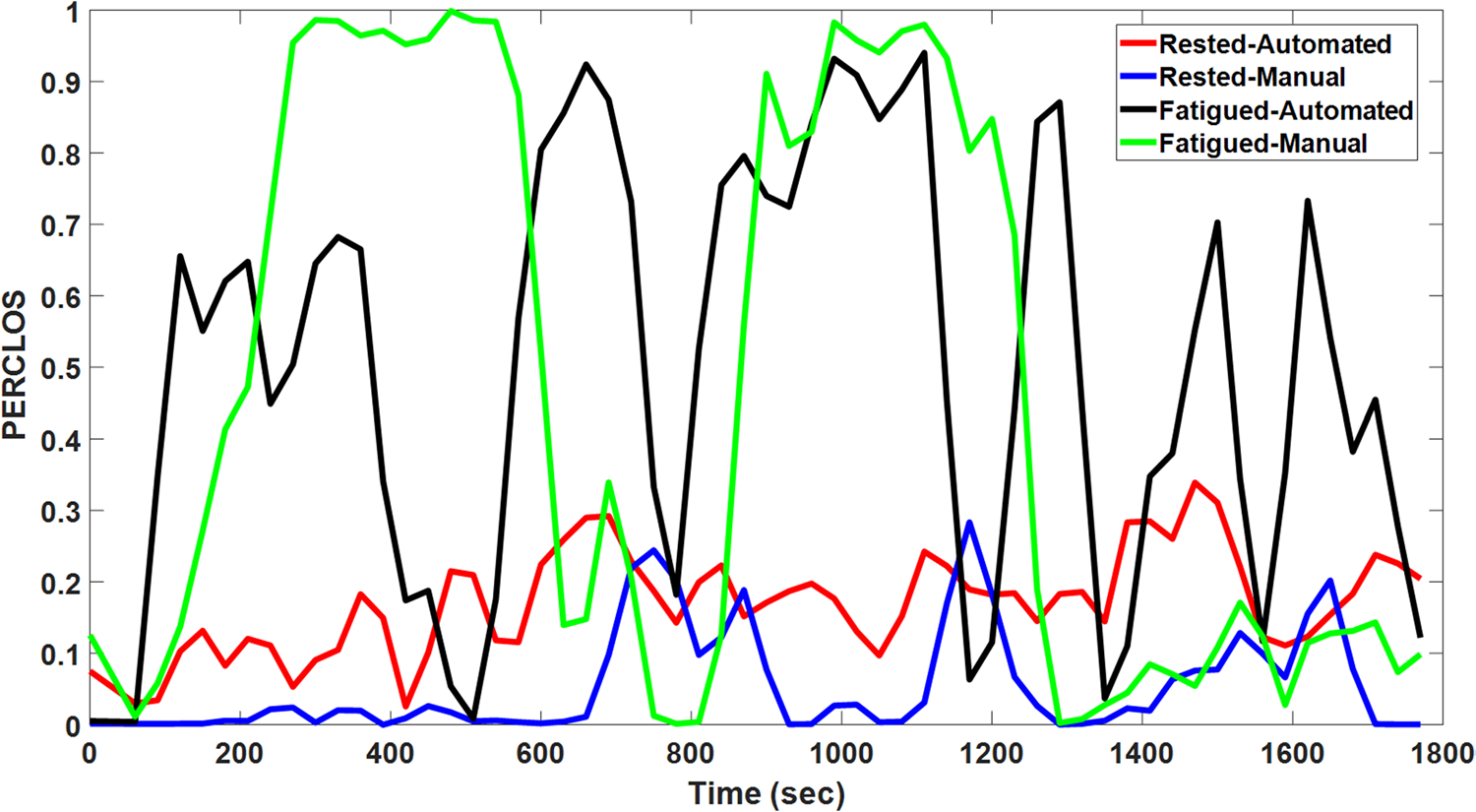
PERCLOS data of four tests in different driving modes: rested-manual, rested-automated, fatigued-manual, and fatigued-automated. A sliding window with a 1-minute length and 30 s overlap between every two adjacent time windows has been used to compute PERCLOS from the eyelid signal.

PERCLOS is a measure of drowsiness that is defined as the proportion of time in a minute that eyes are at least 80 percent closed^33^. For higher PERCLOS values, where the eyes are mostly closed for longer periods of time than for lower PERCLOS values, subjects exhibit strong correlation with a common sign of drowsiness in driving which is lane deviation in the road^33,40^. To calculate this measure, a one-minute sliding window with a 30 seconds overlap between every two consecutive windows has been applied to the eyelid signal. The PERCLOS of four different driving modes of the same driver is shown in Figure 3. In this Figure, PERCLOS increases up to 0.9 in the Fatigued-Automated test and goes up to 1 (completely closed) in the Fatigued-Manual test. This range of PERCLOS suggests that the driver is extremely drowsy in the fatigued mode tests while PERCLOS is barely higher than 0.3 in the rested tests.

### Preprocessing of the EEG channels

EEG signals are contaminated by different noise sources including eye movements, eye blinks, and muscle activity. Different methods have been proposed by researchers to preprocess the EEG signals. For example, Empirical Mode Decomposition (EMD) was used in^41,42^ that decomposes the EEG signals into a specified number of mode functions that can characterize both of neural activities and muscle artefacts. Cleaned signals were obtained by using the mode functions which present the neural activities. Wavelet-based denoising methods have also been applied in previous studies^43–45^. In those methods, Discrete Wavelet Transform (DWT) is applied to the EEG signals to decompose them to their wavelet coefficients. A threshold filter is applied then to remove the wavelet coefficients which represent the noise and artefacts. Finally, the cleaned EEG signals are reconstructed using the remaining coefficients. Independent Component Analysis (ICA) is also another method that has been widely used to preprocess the EEG channels^46–48^. ICA decomposes the raw EEG signals into independent components, where the source of each component can be identified using its scalp topography. The denoised signals are reconstructed by removing those components that are not originated from the brain lobes^49,50^. In this paper, we applied the ICA method via the EEGLAB toolbox^51^ to preprocess the EEG data. This toolbox provides some visual information using scalp topography that makes it easier than other methods to remove noisy components when the clean data are retained in the reconstructed channels. Supplementary Information presents more details about using this toolbox for EEG preprocessing.

### PERCLOS neural encoder model

**Figure 4:**
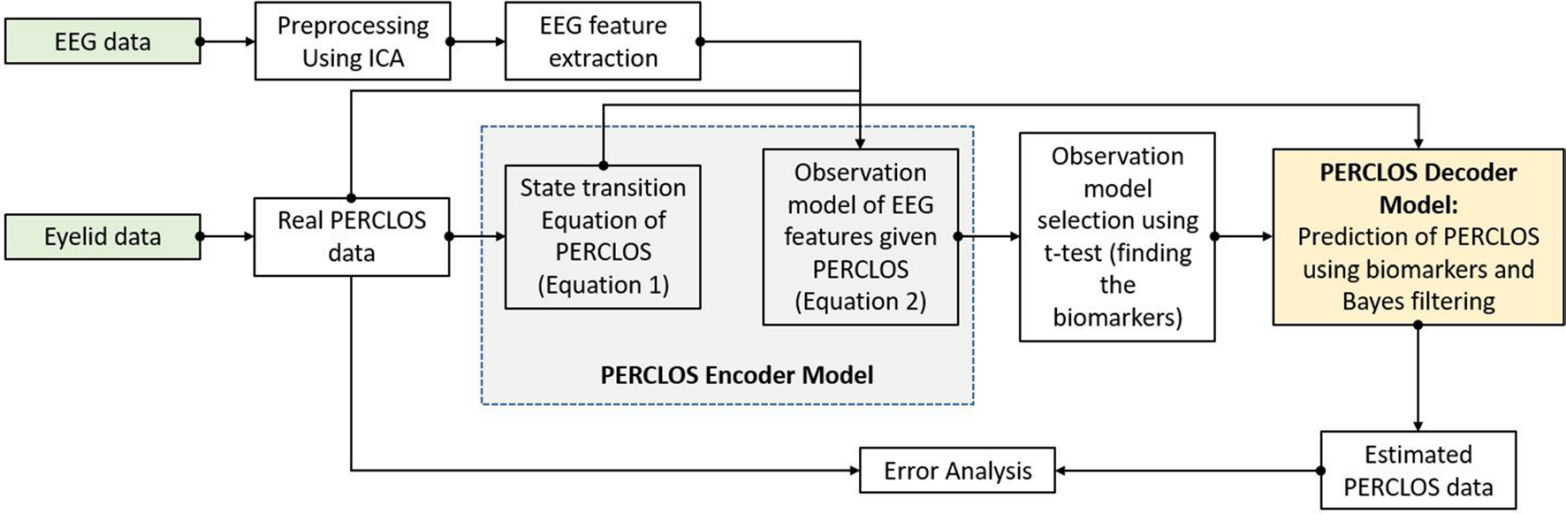
Flowchart of the proposed neural encdoer-decoder modeling framework for PERCLOS. This framework has two main steps: encoder model, and decoder model. In the encoder model, state transition process of PERCLOS and the relationship between every EEG feature and PERCLOS are defined. Biomarkers are obtained using t-test and they are used in the decoder step to predict the PERCLOS in real-time using Bayes filtering.

In this paper, driver drowsiness is considered as hidden cognitive state that cannot be measured or known directly. According to the literature^33,52,53^, we can assume that PERCLOS represents the levels of driver’s vigilance and therefore we might estimate driver vigilance by directly estimating the PERCLOS with an encoder-decoder model that uses EEG features. The flowchart of the proposed framework is presented in Fig. 4. In this subsection, the elements of our proposed encoder-decoder model are discussed. Before building the encoding model, the driving tests are randomly separated into two sets: training set, and test set. Three driving tests with the IDs of 6, 9, and 15 make the test set and the data of the other fifteen tests are used as the training set. The encoder model is designed by using only the training set. The encoder model consists of: (1) a dynamical model to characterize how PERCLOS evolves over time as a state variable independent of EEG data, and (2) an observation model which characterizes how PERCLOS is encoded in the neural features. Each of these models has a separate set of learnable parameters that are estimated using linear regression.

We assume that PERCLOS is a stochastic process characterized by a positive random variable, with a range of 0 to 1. PERCLOS temporal dynamic over-time is defined by the state transition process shown in Eq. (1). This Equation shows our prior knowledge of how PERCLOS changes over-time without knowing the neural activity. As this Equation shows, PERCLOS in every time index only depends on its value in the previous time index and it is independent of the EEG features. We assumed that this equation is valid for the calculated PERCLOS values for all drivers and the PERCLOS of training set tests are concatenated to each other to identify its unknown parameters.

1 $$\begin{aligned} {X}_{i} = 0.5 (1+\tanh (a{X}_{i-1}+b+\epsilon _{i-1}))\; ; i = 1, 2, ..., K \end{aligned}$$

In this Equation, ${X}= [x^{1}, x^{2}, ..., x^{Ntr}]$ is the $1 \times K$ vector of PERCLOS made by horizontally concatenating of the PERCLOS values of the training tests ($x^{1}$ to $x^{Ntr}$) and *Ntr* is the number of driving tests in the training set which here is 15. $\{a,b\}\in \mathbb {R}$ are free unknown parameters and $\epsilon$ is a zero-mean Gaussian noise with the unknown variance of $\sigma _{\epsilon }^{2}$; $\epsilon _{i} \sim \mathcal {N}(0,\,\sigma _{\epsilon }^{2})$. The identification of these unknown parameters is described in the subsection of ”Estimation of state transition process’ parameters”.

We assume that $Y^{d} = [y^{1,d},y^{2,d},...,y^{C,d}]$ is the $L \times C$ matrix of EEG features extracted from the *d*-th driving test of the training set ($d = 1, 2, ..,Ntr$), where *C* is the number of EEG features and *L* is the length of extracted feature and length of PERCLOS vector in every driving test. We also assume that every EEG feature is independent of other features given PERCLOS. Therefore, the conditional distribution of each feature extracted from every driving test, $y^{c,d}$, given the corresponding PERCLOS values of the driver, $x^{d}$, is presented by Eq. (2).

2 $$\begin{aligned} y_{k}^{c,d}|x_{k}^{d}\sim f(x_{k}^{d};\theta ^{c,d})\; ; c = 1,2,...,C\; ; d= 1, 2, ..,Ntr\; ; k = 1,2,...,L \end{aligned}$$

where *f* defines the conditional distribution and $\theta ^{c,d}$ is the set of parameters for the *c*-th EEG feature of *d*-th driving test. Equations (1) and (2) define our dynamical encoder model, characterizing how changes in EEG features over time encode PERCLOS progression in every driving test. In our modeling of the EEG features, we assumed that the conditional distribution of each feature given PERCLOS follows a normal distribution. The mean of the distribution is defined as a linear function of the PERCLOS and the standard deviation of the distribution is assumed to be constant for every EEG feature. This distribution is defined by Eq. (3).

3 $$\begin{aligned} y_{k}^{c,d}|x_{k}^{d}\sim \mathcal {N}(\alpha ^{c,d}x_{k}^{d}+\beta ^{c,d},\,\sigma _{c,d}^{2})\; ; c = 1,2,...,C\; ; d= 1, 2, ..,Ntr\; ; k = 1,2,...,L \end{aligned}$$

where $\alpha ^{c,d}$, $\beta ^{c,d}$ and $\sigma _{c,d}^{2}$ are the unknown slope and intercept parameters and the unknown variance of observed noise for the *c*-th EEG feature of *d*-th driving test, respectively. Therefore, every EEG feature in every driving test has a specific set of parameters given the PERCLOS vector of the corresponding driving test. The Subsection of ”Estimation of observation equation’s parameters” describes the identification of these unknown parameters.

### PERCLOS decoder model

The parameter learning of the method which is based on the training data is performed in the encoding step. The learning process is explained in the “Model Identification” section. However, in the decoder step, the learnt parameters are used in the structure of the Bayesian filtering to estimate the PERCLOS. In other words, the model parameters are not fixed or pre-known and they are estimated using the training data in the encoder step and the same estimated values are used for the decoding step.

**Figure 5:**
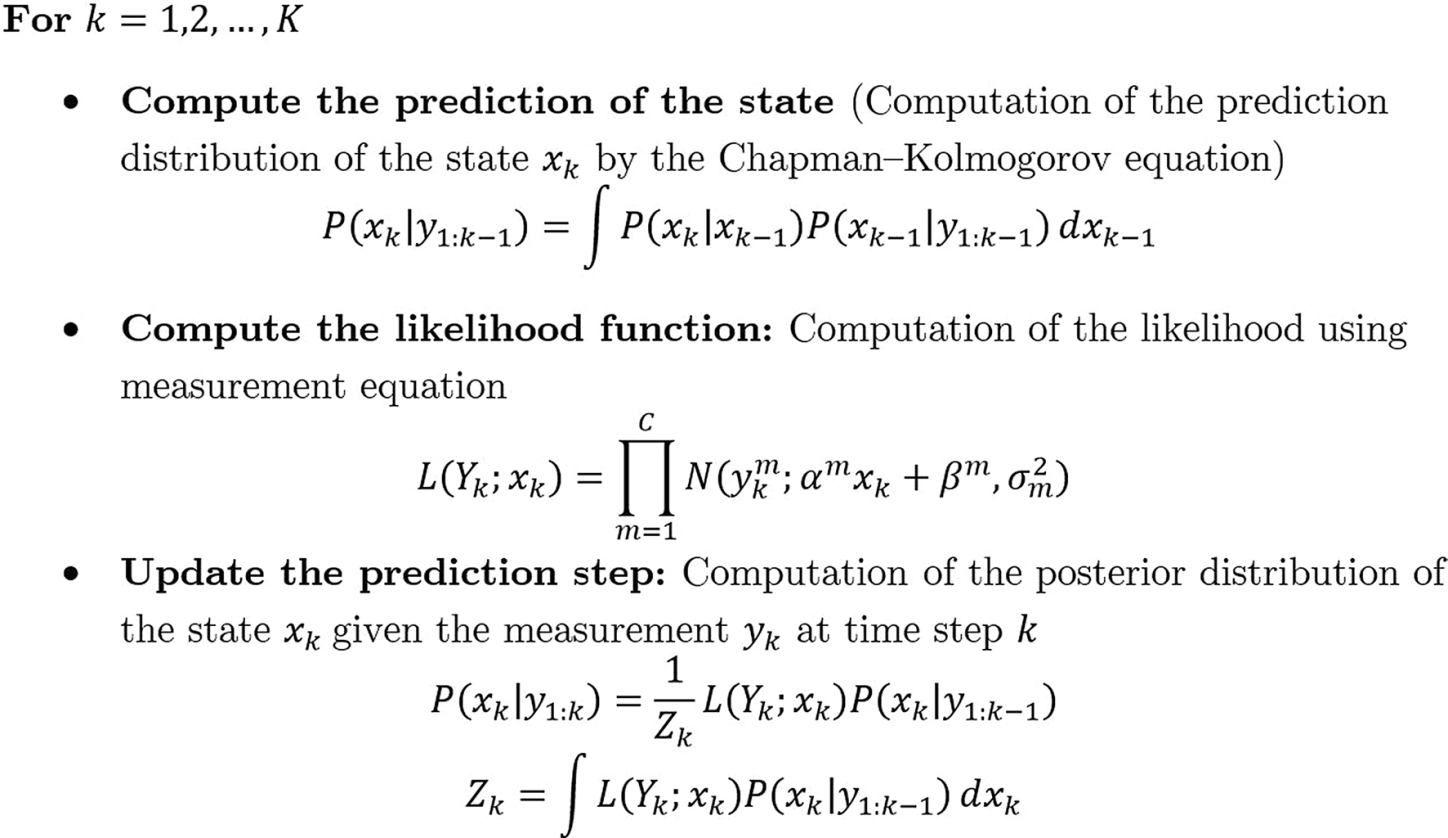
PERCLOS Decoder Model. These three steps are called recursively over time as new neural features are becoming available.

Given the encoder model with estimated parameters, we can use Bayesian filtering to estimate PERCLOS from neural data. This filter provides the best estimation of the PERCLOS, which is our dynamic state, given current and previous values of EEG features through its posterior distribution. The Bayesian filter is a recursive technique that can be conducted by calculating two equations per each time index: one-step perdition and update^54^. Bayesian filter benefits from two processes: state-transition process and observation processes that we have defined these processes in Equations (1) and (3), respectively. Figure 5 demonstrates the general structure of the Bayesian filtering that has three main steps: (1) Chapman-Kolmogorov equation that calculates the one-step prediction of the state, (2) Likelihood function that calculates the likelihood of possible values of PERCLOS given the observed neural feature, and (3) The Bayes’ rule to update the one-step prediction based on the current EEG features. This step updates the filter and provides a posterior distribution of the state (PERCLOS) given the measured observations (EEG features).

### Model identification

In the previous subsections, we described how the encoder model is defined. We also described the decoder or PERCLOS estimation using neural data. For the decoder step, we assumed that the encoder and the state transition model parameters are known; here, we describe how those parameters can be identified.

#### Estimation of state transition process’ parameters

The state transition process defined in Equation (1) can be rewritten by Equation (4). Now, the equation becomes a linear function of *a* and *b* when PERCLOS values are known.

4 $$\begin{aligned} a{X}_{i-1}+b+\epsilon _{i-1} = tanh^{-1}(2{X}_{i}-1)\; ; i = 1, 2, ..., K \end{aligned}$$

where *a*, *b*, and noise variance $\sigma _{\epsilon }^{2}$ are estimated using Least Square (LS) technique. In the LS, it is assumed that $h_{i}=tanh^{-1}(2{X}_{i}-1)$ is the input for the regression problem:

5 $$\begin{aligned} a{X}_{i-1}+b+\epsilon _{i-1} = h_{i}\; ; i = 1, 2, ..., K \end{aligned}$$

The Residual Sum of Square (RSS) error, as presented in Equation (6), is minimized to obtain the parameters of *a* and *b*:

6 $$\begin{aligned} RSS_{X} = \sum _{i=1}^{K} \epsilon _{i-1}^2 = \sum _{i=1}^{K} \left( h_{i}-a{X}_{i-1}-b\right) ^2, \end{aligned}$$

In order to minimize $RSS_{X}$, the derivatives of the $RSS_{X}$ w.r.t two parameters of *a* and *b* are set to be zero:

7 $$\begin{aligned} \frac{\partial RSS_{X}}{\partial a} = 0\; ; \frac{\partial RSS_{X}}{\partial b} = 0 \end{aligned}$$

After applying the Eq. (7) and simplification of the results, the parameters of *a* and *b* are calculated as presented in Eq. (8)^55^.

8 $$\begin{aligned} a = \frac{\sum _{i=1}^{K} \left( X_{i-1}- \mu _{X}\right) \left( h_{i}-\mu _{h}\right) }{\sum _{i=1}^{K}\left( X_{i-1}-\mu _{X}\right) ^2}\; ; b = \mu _{h}-a\mu _{X}, \end{aligned}$$

where $\mu _{X}= \frac{1}{K}\sum _{i=1}^{K} X_{i}$ and $\mu _{h} = \frac{1}{K}\sum _{i=1}^{K} h_{i}$ are the PERCLOS mean and input mean (see Equation 5), respectively. The noise variance is also calculated as the variance of $\epsilon _{i-1} = h_{i}-aX_{i-1}-b$ $(i = 1, 2, ..., K)$.

#### Estimation of observation equation’s parameters

We use linear regression to identify the parameters of the observation equation per EEG feature in every driving test $(\alpha ^{c,d}$, $\beta ^{c,d}$ and $\sigma _{c,d}^{2})$. Linear regression minimizes the root mean square error between estimated and actual EEG features given PERCLOS data. In order to estimate these parameters, Eq. (3) is rewritten as Eq. (9).

9 $$\begin{aligned} y_{k}^{c,d} = \alpha ^{c,d}x_{k}^{d}+\beta ^{c,d}+v_{k}^{c,d}\; ; c = 1,2,...,C\; ; d= 1, 2, ..,Ntr\; ; k = 1,2,...,L \end{aligned}$$

where $v_{k}^{c,d} \sim \mathcal {N}(0,\,\sigma _{c,d}^{2})$ is the Gaussian noise of linear regression of Eq. (9) which is calculated per EEG feature in every driving test. In order to estimate these parameters, the RSS error ($RSS_{y}^{c,d}$) is calculated for each EEG feature in every driving test by using the Eq. (10).

10 $$\begin{aligned} RSS_{y}^{c,d} = \sum _{k=1}^{L} \left( y_{k}^{c,d} -\alpha ^{c,d}x_{k}^{d}-\beta ^{c,d}\right) ^2\; ; c = 1,2,...,C\; ; d= 1, 2, ..,Ntr\; ; k = 1,2,...,L \end{aligned}$$

After minimization of $RSS_{y}^{c,d}$, the unknown parameters of $\alpha ^{c,d}$ and $\beta ^{c,d}$ are determined using Equation (11)^55^.

11 $$\begin{aligned} \alpha ^{c,d} = \frac{\sum _{k=1}^{L} \left( x_{k-1}^{d} - \mu _{x}^{d}\right) \left( y_{k}^{c,d}-\mu _{y}^{c,d}\right) }{\sum _{k=1}^{L}\left( x_{k-1}^{d}-\mu _{x}^{d}\right) ^2}; \; \beta ^{c,d} = \mu _{y}^{c,d}-a\mu _{x}^{d} \; ; c = 1,2,...,C\; ; d= 1, 2, ..,Ntr\; ; k = 1,2,...,L \end{aligned}$$

where $\mu _{x}^{d} = \frac{1}{L}\sum _{k=1}^{L}x_{k}^{d}$ and $\mu _{y}^{c,d} = \frac{1}{L}\sum _{k=1}^{L}{y}_{k}^{c,d}$ and the variance of the error for every EEG feature in every driving test is calculated as the variance of $v_{k}^{c,d} = {y}_{k}^{c,d}-\alpha ^{c,d}x_{k}^{d}-\beta ^{c,d}$.

We also applied some transformations such as logarithm and exponential functions to the EEG features to check if these transformations can improve the regression or not. The autocorrelation of residual errors and R2 coefficient are also calculated to evaluate the goodness of fit for every EEG feature.

### Observation model selection

Though all the neural features can be used in the decoding step, a more practical approach would use only a subset of features that shows strong encoding properties. This process helps to build a more robust and generalizable decoder model by excluding those features which lack reliable and consistent predictive power. With the independence assumption of the neural features, we can check the statistical significance of encoding power of each feature by examining the value of $\alpha ^{c,d}$ described in Eq. (3).

Our null hypothesis is that $\alpha ^{c,d}$ (the slope parameter in Eq. (4)) is zero. Therefore, a t-test per each neural feature has been applied to check whether the null hypothesis can be rejected or not. The* p*-value of 0.05 has been set as a threshold and only those neural features that their corresponding $\alpha ^{c,d}$ comes with strong evidence to fall in the alternate hypothesis, have been selected. This subset of features is then used in the decoding step. Therefore, in the decoding step, only a subset of neural features will be picked, whose statistical significance is in a favor of being included in the encoder model feature set.

## Application of the methodology

In this section, we first discuss how neural features are extracted from EEG signals; we then use the encoder-decoder pipeline to predict the PERCLOS. Preprocessed EEG data of each channel is first decomposed into four sub-bands using band-pass filtering: delta (0.5–4 Hz), theta (4–8 Hz), alpha (8–12 Hz), and beta (12–30 Hz)^56,57^. Then, statistical features of these sub-bands such as their spectral power have been employed in different applications to reduce the dimensionality of the EEG data while significant information is retained during feature extraction^58,59^. These features help to investigate the changes in the EEG data in an interpretable way when the driver drowsiness level is fluctuating during the driving test. Here, fifty features are extracted from each one of the eight EEG channels and one EOG channel that results in 450 neural features for each driving test. The extracted features are listed in Supplementary, Table S1. The same sliding time window that is used to calculate the PERCLOS (1-minute length with a 30-second overlap between two adjacent time windows) has also been applied for EEG feature extraction. All parts of the proposed method have been programmed in MATLAB R2021a. Moreover, the EEGLAB toolbox (v14.1.2) was used in MATLAB to preprocess the EEG data using the ICA technique.

**Figure 6:**
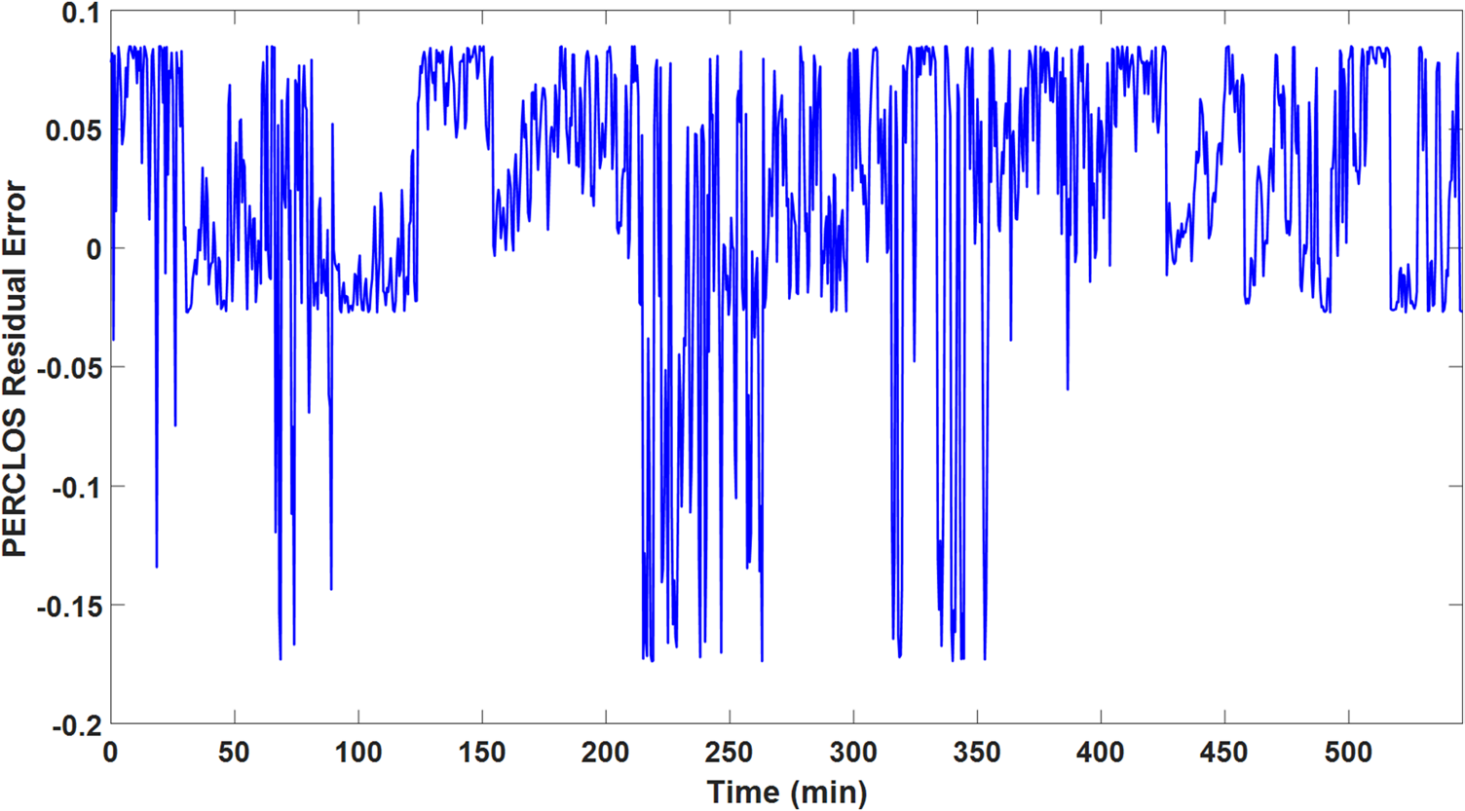
The PERCLOS residual error of the LS method to identify the parameters of the state transition equation. The root mean squares error (RMSE) between actual PERCLOS and modeled PERCLOS by state transition equation is 0.061. This error is higher in some parts of the data which are corresponding to either extremely low (alert) or extremely high PERCLOS (very drowsy) which are not of interest of this method since we want to track the transition of driver’s vigilance from alertness to drowsiness.

To find the state transition process parameters, we concatenated PERCLOS data across all users (resulted in approximately 540 minutes of driving) and use the LS approach to estimate *a*, *b*, and $\sigma _{\epsilon }^{2}$ parameters. In this work, it has been assumed that all users have a reasonably similar state transition process. Therefore, the same estimated parameters of the state transition process (Eq. 1) are used in the Bayesian filtering to estimate PERCLOS. Table 1 represents the estimated parameters for PERCLOS dynamical model defined by Eq. (1). The dynamics of the estimated PERCLOS are adjustable by changing these parameters. For instance, if each of the *a* and *b* parameters approaches the positive infinity, the limit of PERCLOS is one (its maximum value). On the other hand, the limit of the PERCLOS is zero (its minimum value) if each one of these parameters approaches negative infinity. Figure 6 shows the PERCLOS residual error of the outputted result from the LS method using the estimated parameters of the state transition equation. As Fig. 6 shows, the absolute value of the residual error in some parts is about two to three times larger than other parts. After checking the actual PERCLOS values, we realized that these parts are either associated with very high (approximately one) or very low (approximately zero) actual PERCLOS values that show the states of completely alert and extremely drowsy situations, respectively. However, we are aimed to model the dynamic transition between these two states (completely alert and extremely drowsy). The root mean squares error (RMSE) between actual PERCLOS and modeled PERCLOS is 0.061. This result suggests that the proposed state transition process (Equation 1) can reasonably capture the PERCLOS dynamics with acceptable performance.

**Table 1:** State transition process model parameters. These parameters are estimated using LS method and they are assumed to be constant for all of the driving tests. In other words, state transition process of PERCLOS (Equation 1) has the same parameters in different driving tests.

Parameter	Meaning	Value
$\sigma _{\epsilon }^{2}$	Noise variance of state transition process model	0.03
*a*	The slope of the linear regression in Equation (4)	3.93
*b*	The intercept of the linear regression in Equation (4)	−1.79

In the encoder model, a subset of EEG features has been selected using the model selection approach. Note that the decoder model combines two sources of information at different temporal scales in the prediction of PERCLOS: (1) long-term information that is carried by the state process Eq. (1), and (2) instantaneous information carried by neural activity about PERCLOS (Eq. 3). These two sources of information are combined through Bayesian Filtering in the estimation of PERCLOS.

Using the proposed modeling framework, we build a user-specific encoder and decoder model of PERCLOS. we only assumed that PERCLOS temporal dynamics across users share the same characteristics. Given this model, it is possible that a neural feature might be positively correlated with PERCLOS in one participant and negatively correlated in another one. Whilst this might provide a more accurate prediction given the specificity of the model to a specific user, we can search for possible neural biomarkers which are showing consistent encoding properties across participants. We can benefit from the encoding step in the search for possible biomarkers, those that are representing PERCLOS changes consistently across users.

## Results

### Results of biomarker identification

This subsection explains the results of searching the EEG features to find biomarkers of drowsiness. We searched across all EEG features to identify strong correlations to PERCLOS based on their slope ($\alpha ^{c,d}$ in Equation 9). According to the results of the encoder model, 28 highly performant EEG features have been identified that generalize for all 18 driving tests. This means that regardless of the user, these features are significantly realted to the PERCLOS values recorded during the driving test. Therefore, they have the potential to be biomarkers of drowsiness, with highest performance when considered together. These features are presented in Table 2 that include skewness of Alpha of all EEG channels and one EOG channel (9 features), Delta power of all EEG channels and one EOG channel (9 features), Theta power of all EEG channels except Cz and P08 (7 features), and Hjorth mobility of Delta of T8, P08, and EOG channels (3 features).

**Table 2:** EEG features that are consistently significant (*p*-value $< 0.05$) across all the 18 studied driving tests. Overall, 28 features are selected by the encoder regardless of driving tests to encode the PERCLOS dynamics. These features include skewness of Alpha (all EEG channels), Delta power (all EEG channels), Theta power (all EEG channels except Cz and P08), Hjorth mobility of Delta (T8, P08, and EOG channels).

Feature	Channel	Number
Skewness of Alpha	Cz, Fz, T7, T8, C3, C4, P07, P08, EOG	9
Delta power	Cz, Fz, T7, T8, C3, C4, P07, P08, EOG	9
Theta power	Fz, T7, T8, C3, C4, P07, EOG	7
Hjorth Mobility of Delta	T8, P08, EOG	3
—	Sum	28

**Figure 7:**
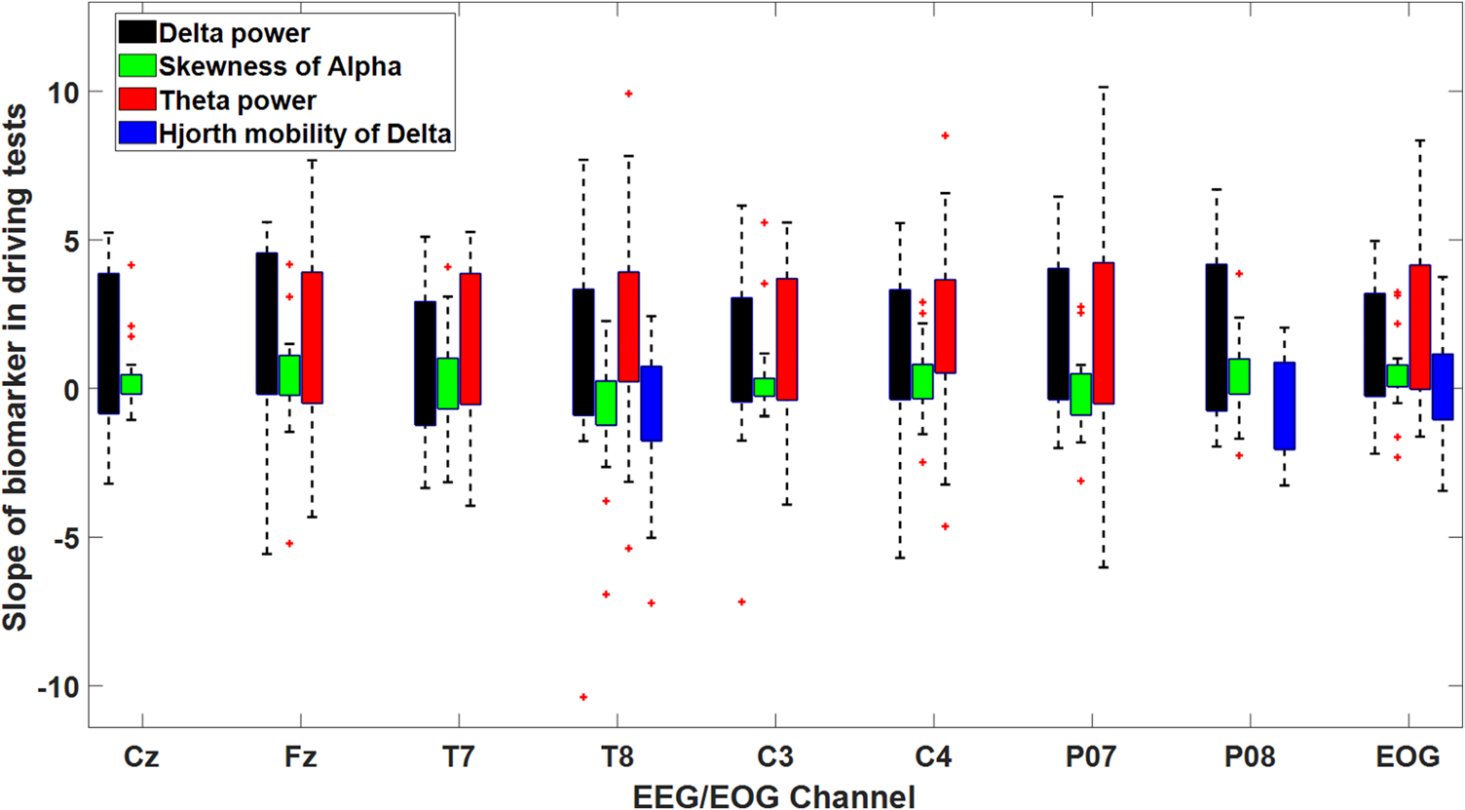
Representation of the slope of the biomarkers in the driving tests. The average values of the Delta and Theta powers of EEG channels are positively correlated with PERCLOS in all of the driving tests.

**Figure 8:**
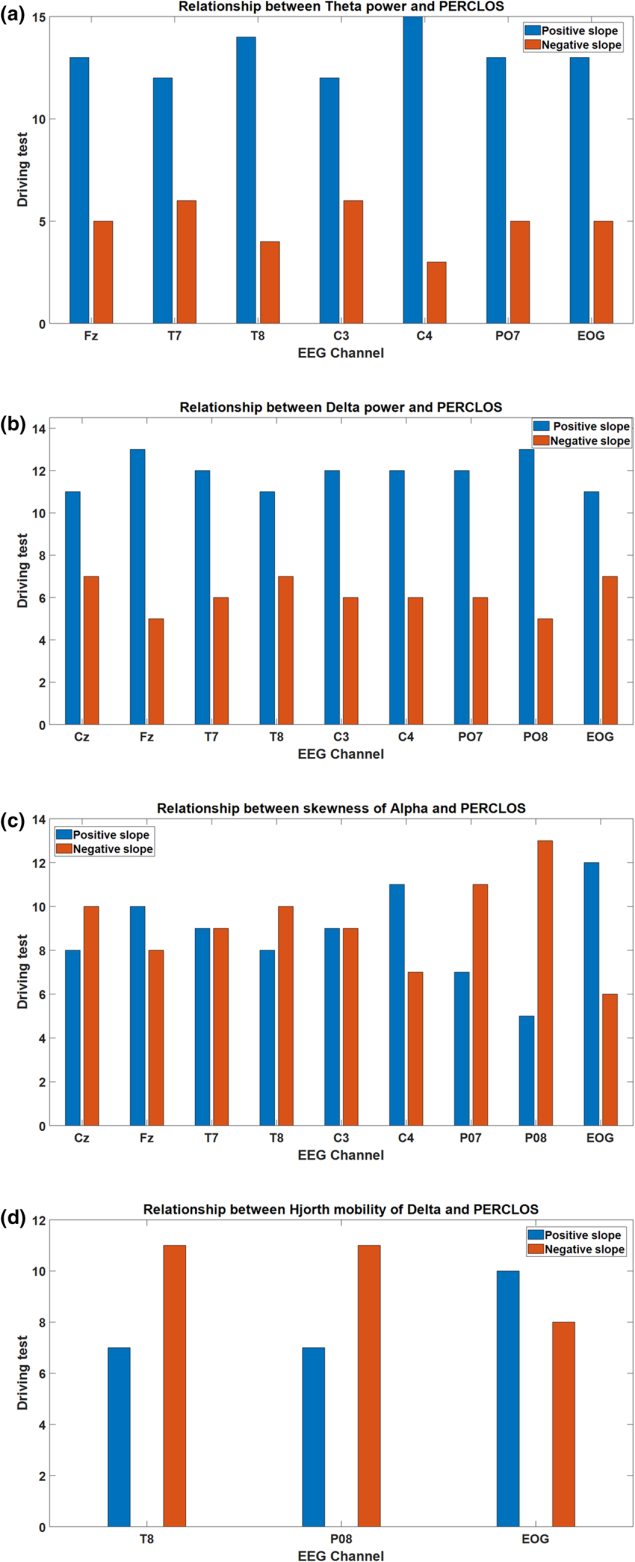
Slope signs of the biomarkers in different driving tests that are statistically significant. According to these results, discovered biomarkers make a ‘push-pull mechanism’ to estimate the driver drowsiness.

Figure 7 also shows the regression coefficient between every biomarker and PERCLOS in all driving tests. As this Figure shows, the average consistent Delta and Theta powers are positively correlated with PERCLOS in all EEG channels. This result is in accordance with established studies that report increases in Theta and Delta powers as indicators of drowsiness^4,58,60^. The skewness of Alpha in all EEG channels except T8 and P07 is also positively correlated with PERCLOS while Hjorth Mobility of Delta in T8 and P08 are negatively and in EOG channels positively correlated with PERCLOS. Therefore, the proposed framework establishes biomarkers that have consistent relationships with PERCLOS. These neural features could therefore be extracted from EEG signals to estimate the drowsiness independently of the drivers and driving conditions. Figure 8 shows the sign of the correlation coefficient of biomarkers and PERCLOS in the driving tests. According to this Figure, about 73% and 66% of the Theta and Delta powers which were selected as biomarkers are positively correlated with PERCLOS, respectively. On the other hand, only 48.8% and 44.4% of the skewness of Alpha and Hjorth mobility of Delta are positively correlated with PERCLOS, respectively. Therefore, discovered biomarkers make a “push-pull mechanism” to estimate the driver drowsiness. In this mechanism, one group of biomarkers that includes Theta and Delta powers are increasing with increasing the level of drowsiness (pushing part of the mechanism), whereas another group of biomarkers that consists of skewness of Alpha and Hjorth mobility of Delta are decreasing with increasing the drowsiness level (pulling part of the mechanism). This interaction between these two parts of the mechanism suggests that considering these biomarkers together provides the best performance for estimating PERCLOS and driver drowsiness and obtains a satisfying estimation of driver drowsiness associated with the PERCLOS data.

### Results of the decoder model for PERCLOS estimation

**Figure 9:**
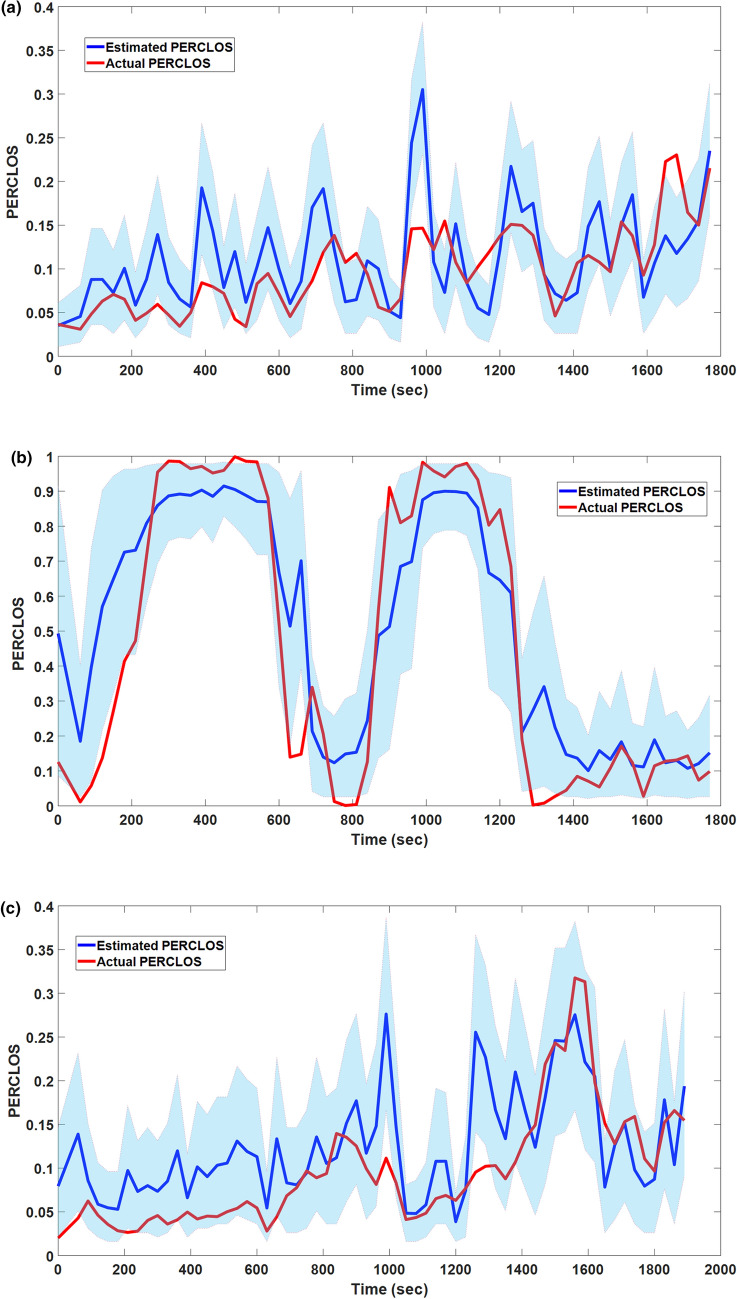
Decoding results in three driving tests with ID=6 (**a**), ID=9 (**b**), and ID=15 (**c**) for estimation of PERCLOS using selected EEG features. Light blue shaded areas show the 95% confidence interval of the estimated PERCLOS. The result suggests a strong correspondence between measured PERCLOS and estimated one.

In this subsection, we discuss the modeling results of our proposed encoder-decoder framework in the estimation of PERCLOS. The data set has been randomly separated into two data sets: train and test. The training set contains 15 tests where three driving tests with ID = 6, 9, and 15 have been selected to make a test dataset. The test dataset has not been involved for selecting the biomarkers. Neural biomarkers (see Table 2) are found using training dataset and are employed to estimate the PERCLOS in the test dataset. In Fig. 9, we show the decoding results of the test dataset. These results suggest that the proposed framework reasonably traces the drowsiness level presented by the actual PERCLOS data. Figure 9 also presents the upper and lower bounds of the 95% confidence interval of the Bayesian estimation. These bounds are utilized to calculate the High Probability Density (HPD) percentage^61^. The HPD presents the percentage of the data samples per driving test where the actual PERCLOS falls in the 95% confidence interval of the estimated one.

**Figure 10:**
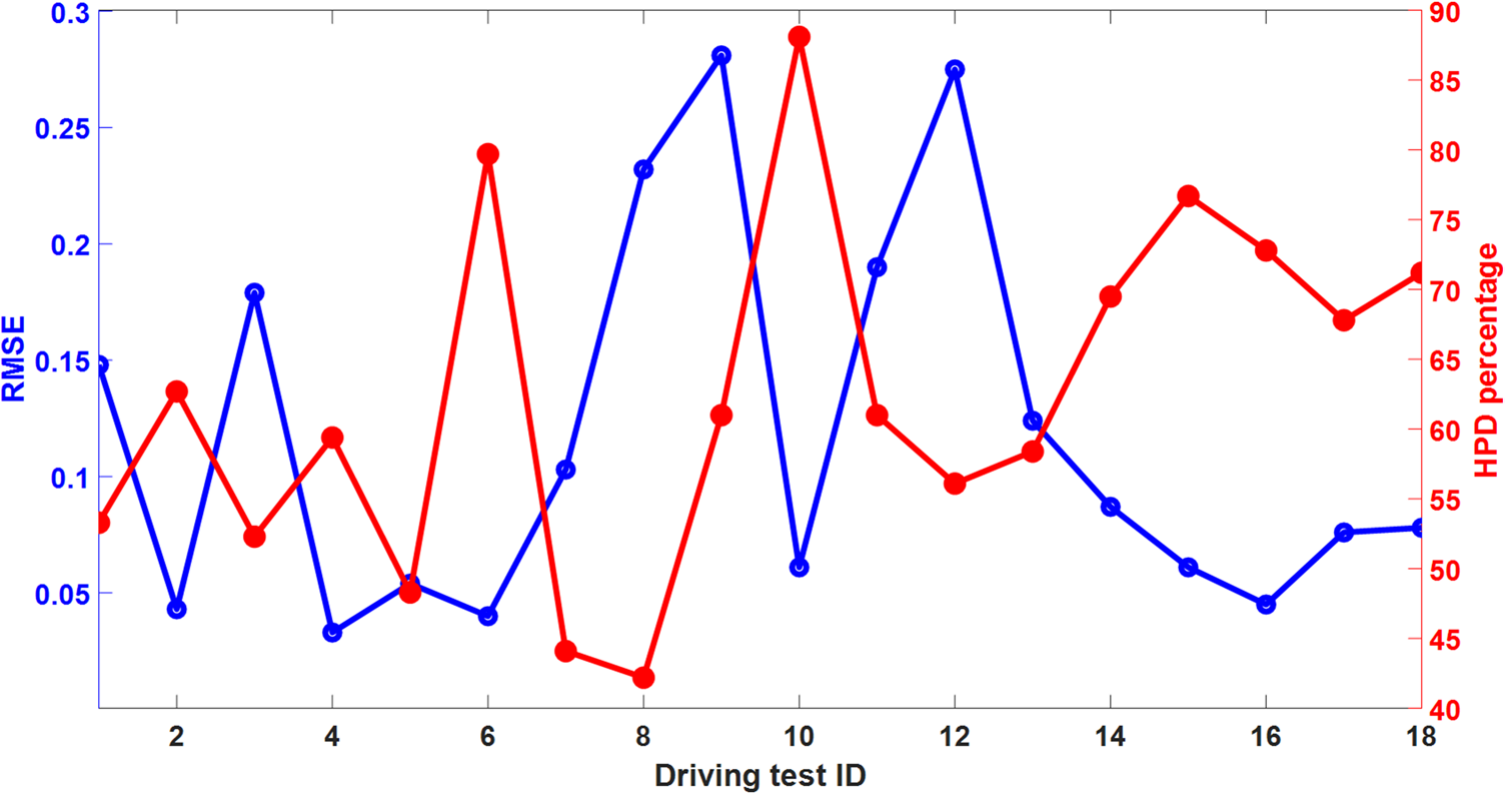
RMSE and HPD percentage metrics to evaluate the performance of the proposed encoding–decoding framework. The average RMSE and average HPD percentage across different driving tests are 0.117 and 62.5%, respectively.

**Figure 11:**
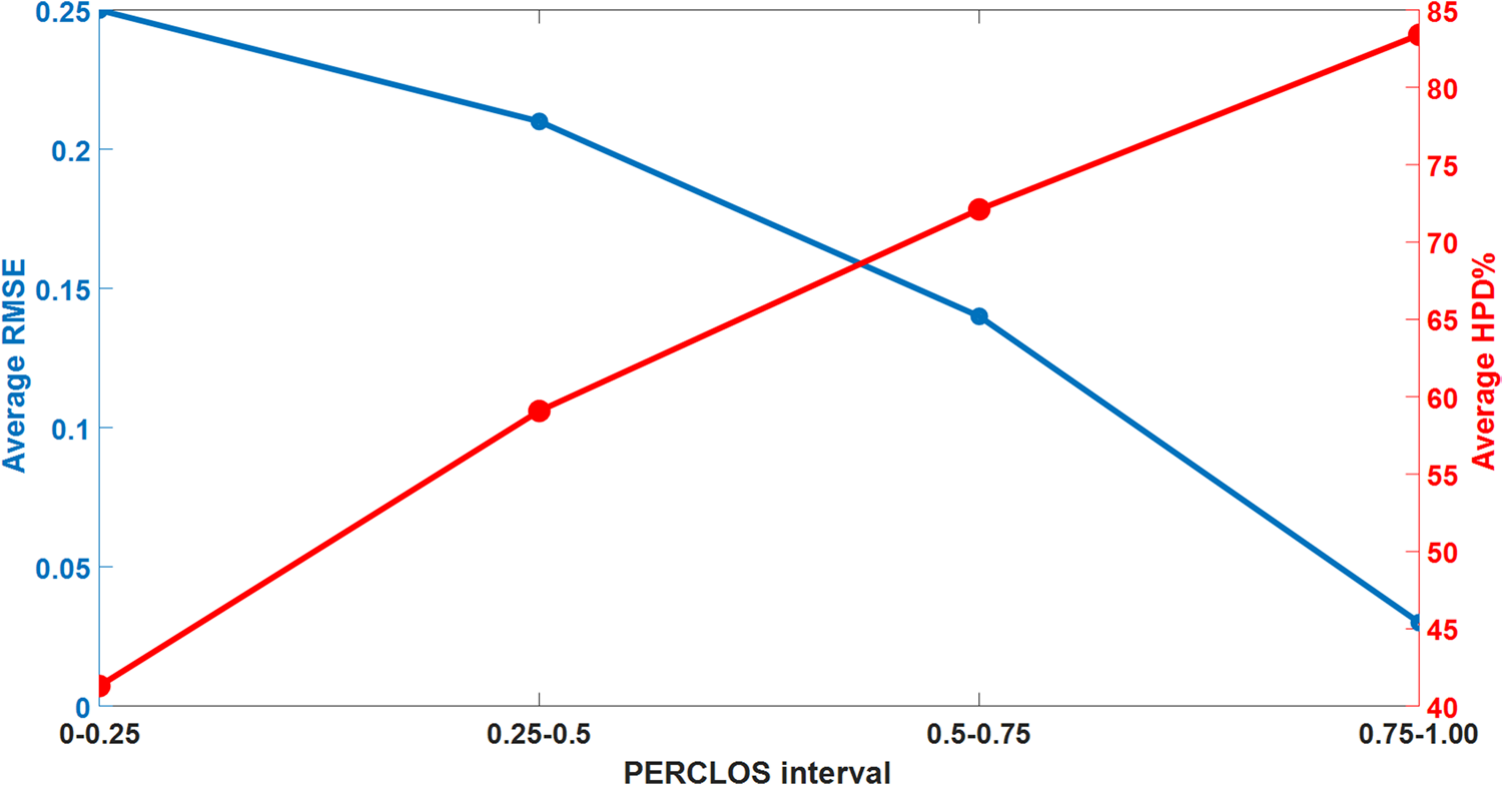
The average RMSE and HPD percentage in different PERCLOS intervals. This Figure shows that the proposed method is performing better in higher values of PERCLOS that are more important to detect the moderate and extreme levels of driver drowsiness. Tests with the IDs of 6, 9, and 15 are used as test dataset.

In order to investigate the PERCLOS estimation accuracy of the frameworks, the RMSE and HPD percentage metrics for PERCLOS estimation are provided in Fig. 10 for all driving tests. This Figure shows that the average RMSE and average HPD percentage are 0.117 and 62.5%, respectively. Moreover, to study the performance of the method in the different levels of drowsiness, PERCLOS is separated into four intervals: 0-0.25, 0.25-0.5, 0.5-0.75, and 0.75-1. The average RMSE and HPD percentage of each one of these intervals during all of the driving tests are presented in Fig. 11. According to this Figure, this average HPD percentage and RMSE are increasing and decreasing as PERCLOS grows, respectively. Therefore, this model obtains better performance in the higher actual PERCLOS (moderate and extreme levels of driver drowsiness), which in practice is more important to detect the driver drowsiness than states with low PERCLOS values.

## Discussion

Different biosignal-based methodologies have been proposed in the literature to estimate the drivers’ workload and their cognitive states^62–64^. For example, electrodermal activity was recorded in^65^ with simultaneous collection of subjective rating of mental workload during driving tests where drivers were asked to perform a time-production task. Results showed that electrodermal activity increases together with subjective ratings in more complex driving scenarios that would indicate higher levels of driving workloads. Argyle et al.^66^ also investigated the relationship between fatigue as a cognitive state and heart rate, breathing rate, and hemodynamic response in the prefrontal cortex as physiological responses. According to the results, fatigue significantly influences physiological responses.

In Brain-Computer Interface (BCI) applications^67,68^ and clinical neuroscience^69,70^, researchers are interested to find neural biomarkers. To find biomarkers, the data of different subjects are usually combined while the individual differences between subjects are ignored. Because of the present individual differences, it can be challenging to find neural biomarkers which are scalable from one person to another one across the group. Most of the similar previous studies used EEG data as inputs to discriminative models to classify levels of drivers’ drowsiness. For example, Li et al.^32^ developed a method for drowsiness estimation using powers of Theta, Alpha, and Beta subbands of EEG data while the ground truth for drowsiness was derived by a combination of PERCLOS and Number of Adjustment (NOA) of the steering wheel^71^ during the test. Considering this ground truth, three classes were defined: (1) alert (PERCLOS$<8\%$ and NOA$>26$), (2) early warning (8%$\le$PERCLOS$<12\%$ and $9<$NOA$\le$26), and (3) drowsy (PERCLOS$\ge$12% and NOA$\le$9). Finally, a support vector machine was used as classifier and according to the results, this method provided classification accuracies of 91.25%, 83.78%, and 91.92% for alert, early warning, and drowsy classes, respectively. A Convolutional Neural Network (CNN) was also applied to the EEG data for drowsiness detection in^72^. In that study, the Alpha-Theta waves (5–9 Hz) of two occipital (O1 and O2) electrodes and two temporal (T7 and T8) electrodes were used as inputs to a Convolutional Neural Network (CNN) network while data augmentation was also used to reduce the risk of over-fitting. Results demonstrated that this method achieved the binary classification accuracy of 90% for driver drowsiness classification. Detection of more levels of drowsiness was also studied in some previous works. For example, the classification of driver drowsiness into five different classes was performed in^73^ using EEG channels and by applying a combination of CNN and Bidirectional Long-Short-Term-Memory (Bi-LSTM) network. In that study, the CNN extracted the features from EEG data and Bi-LSTM derived the long-term dependencies between extracted features. According to the results, this method achieved an average classification accuracy of 69% for five different levels of driver drowsiness.

The main advantage of our proposed method over the previous method is its capability for finding neural biomarkers that consistently encode the drowsiness dynamics in different drivers independent of their characteristics (e.g. age and gender) and driving conditions (manual or automated). Moreover, our method estimates the real-time estimation of drowsiness by providing the estimation for the posterior distribution of PERCLOS. This posterior distribution can also be used to predict the drowsiness level in the next time indices.

In this paper, we proposed a new modeling framework using neural activities to provide an instantaneous estimation of the PERCLOS as a widely used estimation of driver drowsiness. The PERCLOS is being considered as a robust correlate of driver drowsiness which is widely studied to assess driver’s performance in the different vigilance states. Our proposed framework is derived from extensive work in the neuroscience domain where the question was finding the relationship between cognitive state and neural correlates^36,74,75^. The framework has two steps: encoder and decoder. When each of these steps is built through a sequential process, we come up with a dynamical estimation of PERCLOS as a function of the selected number of neural features. One of the advantages of this method compared to previously developed methods is that we are providing a posterior distribution of PERCLOS at every time point which is a fairly complete measure of PERCLOS. Through this measure, we can build other metrics which can be used to assess the trajectory of a driver’s drowsiness and anticipate the time that the driver can be in a dangerous level of driver drowsiness or even decide about whether the driver drowsiness level is above a specific predefined level or not (a predefined threshold might be used to trigger appropriate action or warning). Another advantage of this model is the real-time estimation of driver drowsiness that can reduce the risk of accidents caused by drowsy driving.

Given the preprocessing and encoding strategy of our model, the decoding step for drowsiness estimation requires low computational effort and can be performed in real-time as data is collected. In the preprocessing step, artifacts that might have undesirable effects on the system’s performance are rejected from EEG data, and in the encoding step, the EEG features that can encode the PERCLOS (biomarkers) are discovered across participants. Consequently, only 28 features out of the initial 450 features are used in the decoding step. Therefore, the decoding step is computationally inexpensive and we can have a real-time estimation of PERCLOS. Compared to previously used deep learning methods^76–78^, our proposed method also needs a lower computational cost in the training phase to find out the neural biomarkers. This advantage is obtained by applying the prior knowledge of the real PERCLOS dynamics regardless of EEG features, and also the discovered relationship between EEG features and PERCLOS in the encoder step.

The most important element of our research is the principal approach to find neural biomarkers for driver drowsiness which has not yet been extensively studied in previous works as those works were classifying driver drowsiness as a black-box model^79–81^. In this study, we are providing more details about the relationship between neural activities and PERCLOS in an interpretable manner for the benefit of other researchers in our domain. For instance, we found that the Theta power of the C4 EEG channel is a biomarker of drowsiness which increased with higher PERCLOS values in 15 out of 18 driving tests. On the other hand, the Alpha power of the PO8 EEG channel is another example of obtained biomarkers that decreased in 13 out of 18 tests for higher PERCLOS values.

Although we added new utilities to this domain, more research should be conducted to enhance the performance of this method. Some of the challenges that need to be addressed are as follows:

1. Producing a personalized model is possible by including more EEG channels but there is a trade-off between the utility of the device and the number of EEG channels. We think that rather than increasing the number of EEG channels, more physiological information such as ECG and heart rate variability data^17,82^ can be utilized to enhance the model performance with greater feature independence and robustness.
2. Although the proposed method provides us a solution to find neural biomarkers, we have only studied the fluctuation of nodal frames while more advanced techniques are studying network global dynamics^83,84^. Employing other features that are presenting the network global dynamics of brain activities like coherence, correlation, and mutual information between different EEG channels^85–87^ might improve the performance of the framework. It should be noted that the proposed framework is flexible enough to incorporate those features into our model but the question is which one of them are informative to estimate the driver drowsiness. The proposed framework can also be helpful to select the informative features.
3. This paper discussed the estimation or decoding capability of the proposed framework but another important application of this method is its prediction capability where we can predict what will be the level of drowsiness based on the current and previous neural activities. This capability requires to use a more accurate state transition process that is tuned for every individual driver.
4. In this method, the same estimated parameters in the encoder step were used in the Bayesian filtering of the decoder step to estimate the drowsiness. However, these parameters can also be estimated online in a recursive way when new data are available in every time index. Reinforcement learning can also be used as the method for parameter updating when the driving performance (e.g. reaction time to a traffic event) is used a reference data to provide the award/punishment mechanism.

## Conclusions

One of the requirements of upcoming automated cars is monitoring the driver’s states since he/she is responsible for controlling the car in case of system failure of automated cars. Drowsiness is one of the drivers’ mental states that can significantly degrade driving performance and increase reaction time in critical situations where an accident may be avoided. Therefore, we concentrated on driver drowsiness prediction using EEG signals which were used in previous studies to detect the early stages of drowsiness^19,20^. In order to accomplish this goal, an encoding-decoding framework based on EEG signals was presented to estimate PERCLOS which is a widely used indirect measure of driver drowsiness. This framework is composed of two main steps: encoder and decoder steps. In the encoder step, the relationship between every EEG feature and PERCLOS of the driving test was assumed to be linear with unknown parameters which were estimated using the least-square method. Moreover, the state transition process of PERCLOS regardless of EEG data was defined which has another set of unknown parameters. The least-square approach was also used to estimate this set of parameters.

The training set of EEG data was used to find out a set of EEG features that are significantly correlated with PERCLOS progression for every driving test. To select these features for every driving test, a t-test with a threshold of 0.05 was used to check the magnitude of the slope parameter of every EEG feature in linear regression given the PERCLOS vector. In the decoder step, only EEG features that were selected in the encoder step were used as inputs to a Bayesian filtering to estimate PERCLOS values in real-time. Moreover, the selected feature set for different driving tests was searched to find EEG biomarkers that encode PERCLOS regardless of the driver and driving condition. Overall, 450 features were extracted from EEG data and according to the results, 28 EEG biomarkers were are discovered out of all features. These 28 biomarkers (instead of all 450 features) were used in the decoder step to estimate PERCLOS.

Results of the decoder step show that the proposed method estimates the PERCLOS values with an average RMSE of 0.117 and an average HPD percentage of 62.5% over all driving tests. Therefore, this method not only estimates the drowsiness in real-time but also provides some EEG biomarkers that encode drowsiness. These biomarkers alleviate the required computational power to estimate the onset of the driver’s drowsiness using EEG data independent of driver-specific factors.

## Supplementary Information

Supplementary Information.
