# Supplementary material for: Driver drowsiness estimation using EEG signals with a dynamical encoder–decoder modeling framework
Source: Sci Rep. 2022 Feb 16;12:2650. doi: 10.1038/s41598-022-05810-x (PMC8850607; doi:10.1038/s41598-022-05810-x)
Supplement: Supplementary file 1 — Supplementary Information. [file 41598_2022_5810_MOESM1_ESM.docx]

**Supplementary Information**

**Title: Driver Drowsiness Estimation Using EEG Signals with a Dynamical Encoder-Decoder Modeling Framework**

Sadegh Arefnezhad^1,*^, James Hamet^2,8^, Arno Eichberger^1^, Matthias Frühwirth^3^, Anja Ischebeck^4^, Ioana Victoria Koglbauer^5^, Maximilian Moser^3,6^, Ali Yousefi^2,7^

^1^Institute of Automotive Engineering, Graz University of Technology, Graz 8010, Austria

^2^Neurable Company, Boston, MA 02108, United States

^3^Human Research Institute, Weiz 8160, Austria

^4^Institute of Psychology, University of Graz, Graz 8010, Austria

^5^Institute of Engineering and Business Informatics, Graz University of Technology, Graz 8010, Austria

^6^Chair of Department of Physiology, Medical University of Graz, Graz 8036, Austria

^7^Assistant Professor, Department of Computer Science Worcester Polytechnic Institute, 100 Institute Road, Worcester, MA 01609, United States

^8^He is now with Vistim Labs Company, Salt Lake City, UT 84103, United States

**^*^Corresponding author:**

Sadegh Arefnezhad, Dr., Institute of Automotive Engineering, Graz University of Technology, Inffeldgasse 11, Graz 8010, Austria; Tel: +43 316 873 – 35270; Email: s.arefnezhad@tugraz.at

***EEG Preprocessing***

EEGLAB Matlab toolbox is used to preprocess the EEG raw data. EEG channels are decomposed to their independent components by ICLabel tool implemented in the EEGLAB [1] is used to check which component is originated by brain. ICLabel provides a probability for every component that shows where it is originated from. This probability is calculated based on the spectral analysis and scalp topography map of every component. For example, Figure S1 presents the map topography and assigned label to independent components of the EEG raw data of the driver with ID = 2. Based on this Figure, independent components of number 1 to number 3 are generated by eye movement activities and must be removed to preprocess the EEG channels.


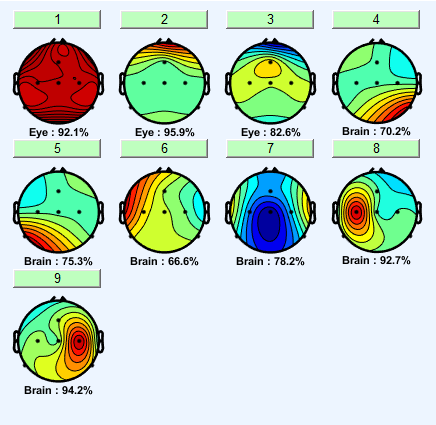


**Figure S1.** Scalp topography maps and assigned labels to every independent component which is obtained as an output of EEGLAB toolbox [1].

Figure S2 shows the one minute of raw and preprocessed Fz channel. As this Figure shows, large EEG amplitudes that are generated by eye movements or blinks are removed in the preprocessed signal.

******

**Figure S2.** Raw and preprocessed EEG Fz channel after removing eye movement components.

***EEG Feature Extraction***

Extracted features from every EEG and EOG channel are listed and Table S1. These features are extracted by applying a sliding time window with a length of 1-min and an overlap of 30-sec between every two consecutive windows.

**Table S1.**  Extracted features from data channels.

| **Index** | **Feature** | **Index** | **Feature** |
| --- | --- | --- | --- |
| *1* | Mean of Delta | *26* | Skewness of Theta |
| *2* | Mean of Theta | *27* | Skewness of Alpha |
| *3* | Mean of Alpha | *28* | Skewness of Beta |
| *4* | Mean of Beta | *29* | Kurtosis of Delta |
| *5* | Standard deviation of Delta | *30* | Kurtosis of Theta |
| *6* | Standard deviation of Theta | *31* | Kurtosis of Alpha |
| *7* | Standard deviation of Alpha | *32* | Kurtosis of Beta |
| *8* | Standard deviation of Beta | *33* | Log energy entropy of Delta |
| *9* | Minimum of Delta | *34* | Log energy entropy of Theta |
| *10* | Minimum of Theta | *35* | Log energy entropy of Alpha |
| *11* | Minimum of Alpha | *36* | Log energy entropy of Beta |
| *12* | Minimum of Beta | *37* | Shannon entropy of Delta |
| *13* | Maximum of Delta | *38* | Shannon entropy of Theta |
| *14* | Maximum of Theta | *39* | Shannon entropy of Alpha |
| *15* | Maximum of Alpha | *40* | Shannon entropy of Beta |
| *16* | Maximum of Beta | *41* | Hjorth mobility of Delta |
| *17* | Median of Delta | *42* | Hjorth mobility of Theta |
| *18* | Median of Theta | *43* | Hjorth mobility of Alpha |
| *19* | Median of Alpha | *44* | Hjorth mobility of Beta |
| *20* | Median of Beta | *45* | Power of Delta $\left( P_{\delta} \right)$ |
| *21* | Mean energy of Delta | *46* | Power of Theta$\left( P_{\theta} \right)$ |
| *22* | Mean energy of Theta | *47* | Power of Alpha$\left( P_{\alpha} \right)$ |
| *23* | Mean energy of Alpha | *48* | Power of Beta$\left( P_{\beta} \right)$ |
| *24* | Mean energy of Beta | *49* | $P_{\beta}/P_{\alpha}$ |
| *25* | Skewness of Delta | *50* | $P_{\beta}/\left( P_{\alpha}+P_{\theta} \right)$ |

***MATLAB Code***

clc,clear all;close all

%% Import the SLMtools

%%% Download it from

%%% "https://nl.mathworks.com/matlabcentral/fileexchange/24443-slm-shape-language-modeling"

%%% add the path to the SLMtools; Edit the folder in the next line

addpath('C:\Users\saref406049\Desktop\EEG_Neurable Paper\MatlabCodesEEG\SLMtools');

%% Load the EEG feature and PERCLOS

%%% The next line imports a struct to the workspace.

%%% This structure has three fields:

%%% (1) code (driving code),

%%% (2) Feature: a struct that includes the features of delta, theta,

%%% alpha, beta, and power ratio features. The order of the features are as

%%% the same as presented in Table S1 in the Supplementary Information file

%%% (3) PERCLOS values in every driving test.

%%% Sliding window: 60 sec, Overlap: 30 sec.

load('EEGFeaturesBands18Tests.mat')

%% Insert the index of the test

prompt = 'Insert the index of the test that you want to process; One number between 1 to 18: ';

indtest = input(prompt);

%% Specify an array for every type of feature and PERCLOS;

%%% Every array would be 3D matrix;

% First dimension: Number of time windows in every test

% Second dimension: Number of features extracted from the subbands; This

% number is 12 for Delta, Theta, Alpha and Beta. Two features are also

% extracted as power ratios: beta/alpha, beta/(alpha+theta);

FeaDel = EEGFeaturesBands18Tests(indtest).Feature.delta; %%% Delta features

FeaThe = EEGFeaturesBands18Tests(indtest).Feature.theta; %%% Theta features

FeaAlp = EEGFeaturesBands18Tests(indtest).Feature.alpha; %%% Alpha features

FeaBet = EEGFeaturesBands18Tests(indtest).Feature.beta; %%% Beta features

FeaRat = EEGFeaturesBands18Tests(indtest).Feature.powerratio; %%% power ratio features

PERCLOS = EEGFeaturesBands18Tests(indtest).PERCLOS; %%% PERCLOS vector of the test

%%% Making the features and PERCLOS of every test to have the same length

L = min(size(FeaRat,1),numel(PERCLOS));

FeaDel = FeaDel(1:L,:,:);

FeaThe = FeaThe(1:L,:,:);

FeaAlp = FeaAlp(1:L,:,:);

FeaBet = FeaBet(1:L,:,:);

FeaRat = FeaRat(1:L,:,:);

PERCLOS = PERCLOS(1:L,:,:);

%%% Constructing the Features Matrix;

Features = [FeaDel,FeaThe,FeaAlp,FeaBet,FeaRat];

%%% Reshaping the Features matrix to have 2D matrix

Features = reshape(Features,[size(Features,1),size(Features,2)*size(Features,3)]);

%%% Normalize the features using zscore

EEGFeaZscore = zscore(Features);

%% Model identification for PERCLOS (state) dynamics

y_tem = atanh(2*PERCLOS+1e-4-1); %% Equation 5 (variable "h") in paper.

%% y_tem = beta0+beta1*PERCLOS+epsilon; Equation 5

beta1 = sum((PERCLOS-mean(PERCLOS)).*(y_tem-mean(y_tem)))/sum((PERCLOS-mean(PERCLOS)).^2); %% "a" in Equation 8

beta0 = mean(y_tem)- beta1*mean(PERCLOS); %% "b" in Equation 8

y_temhat = beta0+beta1*PERCLOS; %%% "h" in Equation 5

PERCLOShat = 0.5*(1+tanh(y_temhat)); %%% Dynamic model of PERCLOS independent of EEG features

%%% Check performance of model identification for PERCLOS dynamics

figure(1)

plot(PERCLOS,'r')

hold on

plot(PERCLOShat,'b')

title('Performance of model identification for PERCLOS dynamics')

%% Calculation the error parameters to be used in the state error distribution

errx = PERCLOS-PERCLOShat; %%% state error independent of EEG features

MSE_x = mean(errx.^2);

RMSE_x = sqrt(MSE_x);

RSS_x = 50*var(errx); %%% varaince of the epsilon in Eq. 4; It is multiplied by 50 to provide a better estimation

%% State process model; Define the distribution of PERCLOS dynamics independent of EEG features.

%%% p_x_x = P(x(k)|x(k-1))

xs = (linspace(0.001,1-0.001,200))';

p_x_x = zeros(length(xs),length(xs));

epxx = [];

for i=1:length(xs)

for j=1:length(xs)

x_1 = xs(i); %%% x(k)---i

x_0 = xs(j); %%% x(k-1)---j

e = atanh(2*x_1+1e-4-1) - beta1 * x_0 - beta0;

p_x_x(i,j) = normpdf(e, 0, sqrt(RSS_x)); %% p_x_x = P(x(k)|x(k-1))

epxx = [epxx;e];

end

end

%%% Normalizing the p_x_x

for j=1:length(xs)

p_x_x(:,j) = p_x_x(:,j)/(sum(p_x_x(:,j))+1e-30);

end

%% Linear fitting for Observation model; Y is every EEG feature; X is PERCLOS

%%% y(k) = a*x(k)+b+v(k);

for i = 1:size(EEGFeaZscore,2)

Fea(i).slm = slmengine(PERCLOS,EEGFeaZscore(:,i),'degree',1,'plot','off','knots',2); %% Linear relationship

Fea(i).yhat = slmeval(PERCLOS,Fea(i).slm,0); %% Calculate the EEG feature using linear relationship

Fea(i).errory = EEGFeaZscore(:,i)-Fea(i).yhat; %% Calculate the error between estimated and real EEG features

Fea(i).R2Adj = Fea(i).slm.stats.R2Adj; %%% R2 Adj parameter of linear fitting

Fea(i).R2 = Fea(i).slm.stats.R2; %%% R2 parameter of linear fitting

Fea(i).NRMSE = Fea(i).slm.stats.RMSE/(max(EEGFeaZscore(:,i))-min(EEGFeaZscore(:,i)));

%%% performing the ttest for error of every EEG feature

[Fea(i).pvalue,Fea(i).durbin] = dwtest(Fea(i).errory,[ones(length(PERCLOS),1),PERCLOS]);

[Fea(i).ttesth,Fea(i).ttestp] = ttest(EEGFeaZscore(:,i),Fea(i).yhat);

end

%% Select the features that their pvalue are higher than 0.05

Indpvalue = [];

for i = 1:length(Fea)

if Fea(i).pvalue>0.05

Indpvalue = [Indpvalue;i]; %% Selected EEG features

end

end

%%% Calculation of linear parameters for every EEG feature; presented in

%%% Equation 9

knot_x = zeros(2,size(EEGFeaZscore,2));

knot_y = zeros(2,size(EEGFeaZscore,2));

C = zeros(size(EEGFeaZscore,2),1);

D = zeros(size(EEGFeaZscore,2),1);

R = zeros(size(EEGFeaZscore,2),size(EEGFeaZscore,2));

for i = 1:size(EEGFeaZscore,2)

R(i,i)= std(Fea(i).errory); %% std of error in Equation 9

knot_x(:,i) = [Fea(i).slm.knots(1);Fea(i).slm.knots(2)];

knot_y(:,i) = [Fea(i).slm.coef(1);Fea(i).slm.coef(2)];

C(i) = (knot_y(1,i)-knot_y(2,i))/(knot_x(1,i)-knot_x(2,i)); %% alpha in Equation 9

D(i) = knot_y(1,i)- C(i)*knot_x(1,i); %% beta in Equation 9

end

C2 = C(Indpvalue); %% alpha in Equation 9

D2 = D(Indpvalue); %% beta in Equation 9

R2 = R(Indpvalue,Indpvalue); % std of error in Equation 9

Measurment = EEGFeaZscore(:,Indpvalue); %% Selected EEG featues for every driving test

%% Bayesian filtering loop

%%% P(x0); Prior knowledge for PERCLOS

p_k_k = normpdf(xs,0.2,1);

M = []; %% mean of estimation

S = []; %% std of estiamtion

pp = []; %% posterior

tt = []; %% likelihood

X_L = []; %% lower bound of HDI

X_U = []; %% upper bound of HDI

for k = 1:length(PERCLOS)

% one step prediction

p_k_k_1 = p_x_x * p_k_k;

% define the likelihood function

L = [];

for i = 1:length(xs)

LL = mvnpdf((Measurment(k,:))', C2*xs(i)+D2, R2);

L = [L;LL];

end

tt = [tt;L'];

%%% posterior

p_k_k = L .* p_k_k_1;

p_k_k = p_k_k/sum(p_k_k);

pp = [pp;p_k_k'];

%%% mean of posterior for PERCLOS estimation

mean_l = sum(p_k_k.*xs);

M = [M;mean_l];

%%% standard deviation of posterior for PERCLOS estimation

std_l = sum(p_k_k.*xs.^2)-mean_l^2;

S = [S;std_l];

%%% Calculate the Upper and lower bounds for High Probability Density

[~,p_ind] = sort(-p_k_k);

p_sum = sort(-p_k_k)*-1;

p_sum = cumsum(p_sum);

[~,t_ind] = min(abs(p_sum-0.95));

sm_l = min(p_ind(1:t_ind));

sm_u = max(p_ind(1:t_ind));

x_l = xs(sm_l);

x_u = xs(sm_u);

X_L = [X_L;x_l];

X_U = [X_U;x_u];

end

%%% Calculate the High Probability Density

hpd_overall = [];

flag_hpd = zeros(1,length(PERCLOS));

for i = 1:length(PERCLOS)

if PERCLOS(i)<X_U(i) && PERCLOS(i)>X_L(i)

flag_hpd(i) = 1;

end

end

%% Ploting the PERCLOS estimation results with lower and upper bounds

hpd_overall = sum(flag_hpd)/length(PERCLOS);

TimePERCLOS = 0:30:length(PERCLOS)*30;

TimePERCLOS(2) = [];

figure()

plot(TimePERCLOS,M,'b','LineWidth',5);

hold on

plot(TimePERCLOS, PERCLOS,'r','LineWidth',5)

plot(TimePERCLOS,X_L, ':', 'color', [.89, 0, .23])

plot(TimePERCLOS,X_U, ':', 'color', [.89, 0, .23])

patch([TimePERCLOS fliplr(TimePERCLOS)], [X_L' fliplr(X_U')], [0, .6, .77], 'FaceAlpha',0.3, 'EdgeColor','none')

legend('Estimated PERCLOS','Actual PERCLOS');

%%% Calculate some estimation error parameters

error_perclos = PERCLOS-M;

RMSE_perclos = sqrt(mean(error_perclos.^2))

NRMSE_perclos = RMSE_perclos/(mean(M))

SSE_perclos = sum((M-PERCLOS).^2);

SST_perclos = sum((PERCLOS-mean(PERCLOS)).^2);

R2_perclos = 1-SSE_perclos/SST_perclos

MAPE_perclos = mean(abs(error_perclos./PERCLOS))*100

**References:**

[1] Delorme, A. & Makeig, S. Eeglab: an open source toolbox for analysis of single-trial eeg dynamics including independent component analysis. J. Neurosci. Methods 134, 9–21, DOI: 10.1016/j.jneumeth.2003.10.009 (2004).
